# Recurrence, Readmission, and Key Mortality Predictors in Patients with Carbapenem-Resistant Enterobacterales Infections

**DOI:** 10.3390/diagnostics15232957

**Published:** 2025-11-21

**Authors:** Bashayer Mohammed Alshehail, Marwan Jabr Alwezzeh, Hussain Humaid Almalki, Amani Alnimr, Haytham Wali, Zainab Al Jamea, Abdullatif S. Al Rashed, Mashael Alhajri, Hawra Abdulwahab Abdulaal, Lujain Ali Alanbari, Yazed S. Alsowaida, Abdullah Alamri, Sharifah Almuthen, Faten Azaiez, Saeed Alzahrani, Nawaf Zakari, Jaber Asiri, Wafa Alanazi, Mohanad Bakkar, Abdulaziz Alfifi, Omar Alzuwayed, Aiman El-Saed, Salma AlBahrani

**Affiliations:** 1 Pharmacy Practice Department, College of Pharmacy, Imam Abdulrahman Bin Faisal University, Dammam 34212, Saudi Arabia; bmalshehail@iau.edu.sa; 2 Infectious Disease Division, Internal Medicine Department, King Fahd University Hospital, Imam Abdulrahman Bin Faisal University, Dammam 31441, Saudi Arabiamhajri@iau.edu.sa (M.A.); 3 Department of Pharmaceutical Care, Northern Area Armed Forces Hospital, Hafar Al Batin 31991, Saudi Arabia; hus.almalkii@gmail.com; 4 Department of Microbiology, College of Medicine, Imam Abdulrahman Bin Faisal University, Dammam 34212, Saudi Arabia; 5 Pharmacy Practice Department, College of Clinical Pharmacy, King Faisal University, Alahsa 31982, Saudi Arabia; hwali@kfu.edu.sa; 6 Pharmaceutical Care Department, King Fahd University Hospital, Imam Abdulrahman Bin Faisal University, Dammam 31441, Saudi Arabia; 7 College of Clinical Pharmacy, Imam Abdulrahman Bin Faisal University, Dammam 34221, Saudi Arabia; hawrabdulaal@gmail.com (H.A.A.);; 8 Department of Clinical Pharmacy, College of Clinical Pharmacy, Hail University, Hail 81442, Saudi Arabia; ys.alsowaida@uoh.edu.sa; 9 Pharmaceutical Service Department, King Fahd Military Medical Complex, Dhahran 34313, Saudi Arabiajaberalkhlaify@gmail.com (J.A.); wafa.anazi@gmail.com (W.A.); alzuwayed020@gmail.com (O.A.); 10 Infectious Disease Unit, Specialty Internal Medicine, King Fahd Military Medical Complex, Dhahran 34313, Saudi Arabiadr.mohanadbakkar@gmail.com (M.B.);; 11 Department of Urology and Nephrology, King Fahd Military Medical Complex, Dhahran 34313, Saudi Arabia; drsalma1@hotmail.com; 12 Internal Medicine Department, King Fahd Military Medical Complex, Dhahran 34313, Saudi Arabia; nawafzakary@yahoo.com; 13 King Abdulaziz Medical City, Riyadh 11426, Saudi Arabia

**Keywords:** carbapenem-resistant Enterobacterales, infection recurrence, mortality, risk factors, Saudi Arabia

## Abstract

**Background**: Carbapenem-resistant Enterobacterales (CRE) are designated by the World Health Organization as critical-priority pathogens. While global outcomes are well documented, regional data from the Middle East remain limited. **Methods:** We performed a retrospective cohort study of adults with confirmed CRE infections admitted to King Fahad Hospital of the University, Saudi Arabia, between 2019 and 2024. Clinical, microbiological, and therapeutic data were analyzed. Primary outcomes were infection recurrence, recurrence-related readmissions, and all-cause mortality at 14, 30, and 90 days. Predictors were assessed using univariate tests and multivariate Cox regression. **Results:** Among 101 patients (mean age 65 years, 57% female), *Klebsiella pneumoniae* predominated (94%), with OXA-48 detected in 70%. Most infections were hospital-acquired (78%). Recurrence occurred in 16.8% of cases, with 12.9% requiring readmission. Mortality reached 22.8% at 14 days, 30.7% at 30 days, and 42.6% at 90 days. Diabetes mellitus predicted recurrence (*p* = 0.024). Independent predictors of 90-day mortality were pneumonia (HR 2.39, 95% CI 1.23–4.64), critical care admission (HR 6.24, 95% CI 2.44–15.97), and hypotension (HR 4.10, 95% CI 1.84–9.15). Elevated Pitt bacteremia and INCREMENT-CPE scores also stratified risk. **Conclusions:** CRE infections in Saudi Arabia impose a heavy clinical burden, with high recurrence, frequent readmissions, and late mortality. Identifying drivers of recurrence and mortality highlights opportunities for targeted risk stratification. Beyond treatment choices, these findings emphasize the need for proactive surveillance, integrated stewardship, and early recognition of high-risk patients. Region-specific evidence such as this is critical to shaping infection control policies and guiding future multicenter research into novel therapeutic approaches.

## 1. Introduction

Carbapenem-resistant Enterobacterales (CRE) have emerged as one of the most critical global health threats, identified by the World Health Organization as a top-priority multidrug-resistant pathogen requiring urgent development of new antibiotics [1]. The global spread of CRE has been driven by multiple factors, including extensive antibiotic use, international travel, and inadequate infection control [2]. CRE exhibit resistance primarily through the production of carbapenemases (e.g., KPC, NDM, OXA-48, VIM, IMP) or a combination of β-lactamase production and porin mutations, often conferring cross-resistance to multiple antimicrobial classes such as fluoroquinolones and aminoglycosides [3].

Clinical outcomes associated with CRE infections are consistently poor. Systematic reviews and multicenter studies have reported mortality rates ranging from 30% to 70% across various infection types [4,5,6], with bloodstream infections particularly fatal [5]. These outcomes are compounded by significant geographic variation. The prevalence of CRE colonization shows global variation, with rates in long-term care facilities reported at 1–30.4% in the United States, 13–22.7% in Asia, and lower rates noted in Europe [7]. In Saudi Arabia, the prevalence ranges from 12% to 32%, with higher rates in intensive care units and tertiary hospitals [8,9].

Reported predictors for CRE mortality include infection type, ICU admission, septic shock, high Pitt bacteremia score (PBS) (≥8), and elevated Charlson comorbidity indices [10,11]. Therapeutic strategies remain controversial. While combination therapy, including colistin-based regimens, may improve outcomes [12], resistance to newer agents such as ceftazidime-avibactam is emerging [13,14], underscoring the need for stewardship-driven optimization.

Beyond mortality, hospital readmission has become a key indicator of patient prognosis and healthcare burden related to CRE infections. Zilberberg et al. and Howard-Anderson et al. reported 30-day readmission rates of 10–30% in large US cohorts, with significant implications for both cost and clinical outcomes. Readmission was also linked to inappropriate empirical therapy and CRE colonization [15,16].

Several additional studies have elaborated on the predictors of readmission and clinical deterioration in CRE cases. For instance, high readmission rates in patients discharged on outpatient parenteral antimicrobial therapy (OPAT) were reported [17]. In addition, the outcomes were poor regardless of CRE subgroup or resistance mechanism [13,18]. Other studies revealed persistent carriage in around 42% of readmitted patients [19,20], further driving healthcare-associated transmission. Risk factors such as short discharge intervals, prior use of fluoroquinolones, and multiple hospitalizations were consistently associated with prolonged carriage of CRE [19].

A multicenter prospective cohort study by Alraddadi et al. [21] conducted across eight tertiary hospitals in Saudi Arabia reported a 30-day mortality rate of over 30% and identified key predictors of mortality, including age, Charlson comorbidity index, Pitt bacteremia score, and infection site. In this study, we examined the key factors predisposing to CRE infection recurrence, recurrence-related readmissions, and associated mortality, as well as the independent predictors of mortality among infected patients. Our findings add region-specific data from Saudi Arabia, enriching the global evidence base on CRE and informing both local and international clinical practice.

This study aimed to identify risk factors for infection recurrence, recurrence-related hospital readmissions, and mortality in patients with carbapenem-resistant Enterobacterales (CRE) infections, and to determine independent predictors of 90-day all-cause mortality in a tertiary care hospital in Saudi Arabia.

## 2. Materials and Methods

### 2.1. Study Design, Settings, and Participants

A retrospective observational cohort study of adult patients with CRE infections was conducted between January 2019 and December 2024 at King Fahad Hospital of the University (KFHU), Khobar, Saudi Arabia—a tertiary care academic hospital in the Eastern Province of Saudi Arabia with a bed capacity of approximately 550 beds.

Patients were included in the study if they were 18 years or older with confirmed CRE infections, whereas patients with CRE colonization, other concurrent infections, or incomplete medical records were excluded.

### 2.2. Definitions

CRE infection was defined as CRE isolated from relevant infection sites and exhibiting signs and symptoms that meet the criteria for the corresponding infection definition. Bloodstream infection was diagnosed in patients with positive blood cultures and related clinical manifestations. Diagnosis of lower respiratory tract infection, urinary tract infection, intra-abdominal infection, and skin and soft tissue infection was based on the IDSA guidelines [22,23,24]. CRE colonization is defined as the isolation of CRE from rectal swabs or other non-sterile samples in the absence of clinical symptoms or signs of infection [14]. Hospital-acquired CRE infection is defined as an infection that occurs after 48 h or more of hospitalization [25].

### 2.3. Study Outcomes

The primary outcomes were the 30-day recurrence rate, recurrence-related hospital admissions, and mortality rates within 14, 30, and 90 days after CRE infection. Secondary outcomes included factors that predispose to CRE recurrence and predictors for 90-day mortality.

### 2.4. Patient Data Collection

The medical records of eligible patients were retrieved and thoroughly reviewed. A structured data collection sheet was used to gather the data, including patient factors such as demographics, comorbidities (e.g., cardiovascular disease, congestive heart failure, chronic lung disease, diabetes mellitus, liver diseases, and immune diseases), and the Charlson Comorbidity Index (CCI). Infection-related information encompasses the types of CRE infections, the onset site of infection—whether community-acquired or hospital-acquired, level of care (e.g., critical care, emergency department, or a regular non-critical ward), length of stay (LOS), previous hospitalization within 12 months, the isolated bacteria, resistance genes, PBS, INCREMENT-CPE mortality score, exposures to antibiotics within the past 3 months, current therapeutic management and duration, and above mentioned outcomes.

### 2.5. Microbiological Data

Bacterial identification was performed using MALDI-TOF MS (VITEK^®^ MS, bioMérieux, Craponne, France). Clinical specimens including blood, urine, respiratory samples, wound swabs, and other sterile site samples were processed according to standard laboratory protocols.

Antimicrobial susceptibility testing (AST) was conducted using the automated VITEK^®^ 2 system (bioMérieux, France) following Clinical and Laboratory Standards Institute (CLSI) guidelines. Carbapenem resistance was defined as resistance to at least one of the following carbapenems: imipenem, meropenem, or ertapenem, based on CLSI breakpoints.

Carbapenemase gene detection was performed using the Xpert^®^ Carba-R assay (Cepheid, Sunnyvale, CA, USA) on the GeneXpert^®^ platform. This real-time PCR-based assay simultaneously detects the five most prevalent carbapenemase gene families: *bla*KPC, *bla*NDM, *bla*VIM, *bla*OXA-48, and *bla*IMP-1. Testing was performed directly from positive bacterial cultures according to the manufacturer’s instructions.

Quality control strains were tested concurrently to ensure accuracy and reproducibility of testing methods, in accordance with CLSI recommendations and manufacturer guidelines.

### 2.6. Statistical Analysis

SPSS (Version 25.0; Armonk, NY, USA: IBM Corp) was utilized for all statistical analyses. Categorical data were presented as frequencies and percentages, while continuous data were presented as mean and standard deviation (SD) or median and interquartile range (IQR), based on data normality. Differences in categorical variables were assessed using the Chi-square test or Fisher’s exact test, as appropriate. Continuous variables were compared with either Student’s *t*-test or Mann–Whitney U test, based on data normality. Demographic and clinical characteristics were analyzed in relation to CRE infection recurrence and mortality. Kaplan–Meier mortality-free survival was run and stratified by type of infection. Differences in survival between groups were examined using the log-rank test. A multivariate Cox regression analysis was performed to identify potential predictors of mortality using a backward elimination approach. The hazard ratios were adjusted for the variables that were significant in univariate analysis. All *p*-values were two-tailed. *p*-value < 0.05 was considered significant.

### 2.7. Ethical Considerations

The study was conducted in accordance with the ethical principles outlined in the Declaration of Helsinki by the World Medical Association. The study was conducted in accordance with the Declaration of Helsinki, and approved by the Institutional Review Board of Imam Abdulrahman Bin Faisal University (IRB-2024-05-340, approval date: 2 May 2024) for studies involving humans. The IRB waived the requirement for informed consent, as patient data were analyzed retrospectively and anonymized.

## 3. Results

**Figure 1:**
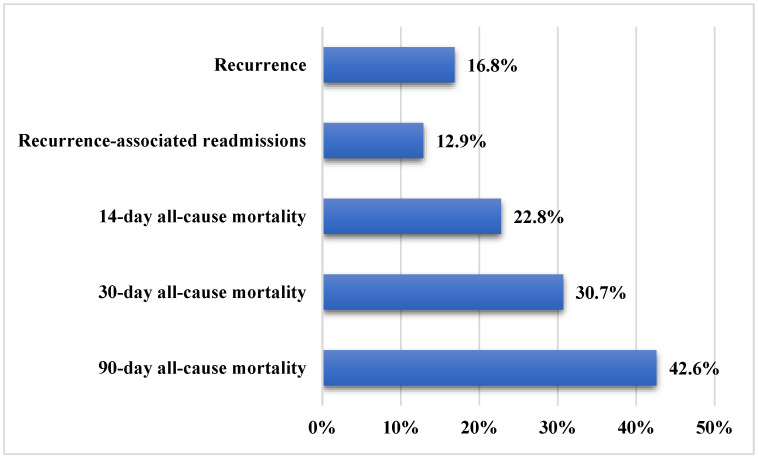
Study outcomes in patients with carbapenem-resistant Enterobacteriaceae infections (*n* = 101).

A total of 101 patients were included in the current analysis. Demographic and clinical characteristics of the patients are summarized in Table 1. Approximately 57.4% of the patients were females, and the mean age was 65.1 ± 17.1 years. The main CRE pathogens were *Klebsiella pneumoniae* (94.1%), followed by *Escherichia coli* and *Serratia marcescens* (5.0% and 1.0%, respectively). The CRE resistance genes were OXA-48, NDM, or both in 70.3%, 10.9%, and 18.8% of the samples, respectively. The most frequent CRE infections were urinary tract infections (35.6%), bacteremia (26.7%), pneumonia (23.8%), and skin/soft tissue infections (8.9%). The source of infection was mainly hospital-acquired (78.2%). Figure 1 presents the study outcomes. Recurrent CRE infection occurred in approximately 16.8% of patients, while readmissions related to recurrence accounted for 12.9% of all readmissions. All-cause mortality rates were 22.8% at 14 days, 30.7% at 30 days, and 42.6% at 90 days of follow-up.

The average Charlson comorbidity index (CCI) was 4.8 ± 2.7, with 55.4% of the patients being at high risk (≥5). 26.5% of the patients were using antibiotic combination therapy, mainly ceftazidime/avibactam (CAZ-AVI) or colistin combinations. On the other hand, 73.5% of the patients were using antibiotic monotherapy, mainly CAZ-AVI (41.8%) and carbapenems (15.3%). The median (IQR) duration of antimicrobial therapy was 12 (7–18) days.

Table 2 and Table 3 show the associations between CRE recurrence and demographic variables, type of CRE pathogens, CRE genes, type of CRE infections, sources of infection, comorbidities, previous hospitalization, previous antibiotic exposures, prolonged LOS, antibiotic therapy regimens, and antibiotic therapy duration. None of the listed variables was significantly associated with CRE recurrence. Diabetes mellitus (DM) was the only comorbidity significantly associated with CRE recurrence (*p* = 0.024).

On the other hand, the following predictors that presented in Table 2 and Table 4 were significantly associated with 90-day mortality; bacteraemia (*p* = 0.001), pneumonia (*p* = 0.006), critical care admission (*p* < 0.001), hypotension (*p* < 0.001), inotropic support (*p* < 0.001), cardiac arrest (*p* = 0.007), altered mental status (*p* < 0.001), continuous renal replacement therapy (CRRT) (*p* = 0.001), mechanical ventilation (*p* = 0.001), and severe sepsis/septic shock (*p* < 0.001). In addition, the Pitt bacteraemia score (PBS) and INCREMENT-CPE score were significantly associated with 90-day mortality, with *p* < 0.001. In contrast, urinary tract infection (46.6% of survivors versus 20.9% of non-survivors, *p* = 0.008) and Colistin-based combination therapy (10.6% of survivors versus 0.0% of non-survivors, *p* = 0.039) were significantly more prevalent in survivors at day 90.

**Figure 2:**
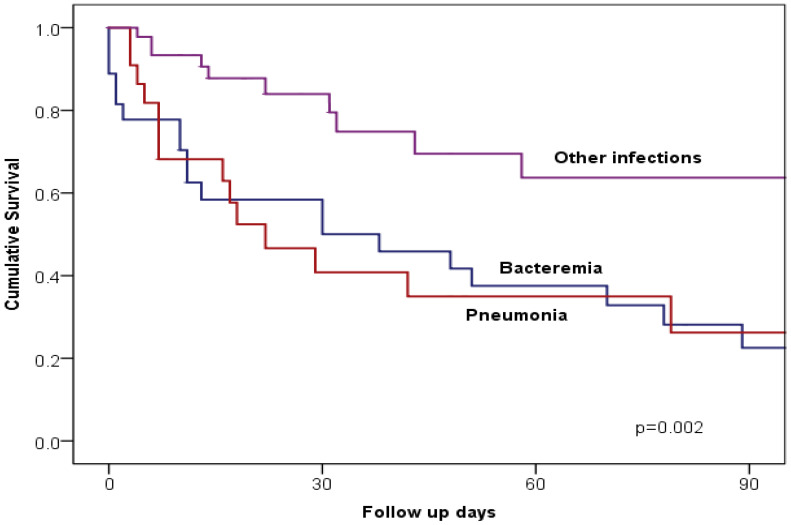
Kaplan–Meier curves illustrate 90-day all-cause mortality in patients with CRE pneumonia, bacteremia, or other CRE infections.

As shown in Figure 2, Kaplan–Meier mortality-free survival among patients with CRE infection was significantly worse among those with bacteraemia or pneumonia compared with those with other types of infection (*p* = 0.002). Table 5 shows the multivariate Cox regression analysis of potential predictors of mortality among patients with CRE infection. After adjusting for the variables that were significant in univariate analysis, the following variables were independent predictors of high mortality; pneumonia (Hazard ratio = 2.39, 95% confidence interval [CI] 1.23–4.64, *p* = 0.010), critical care (Hazard ratio = 6.24, 95% CI 2.44–15.97, *p* < 0.001), and hypotension (Hazard ratio = 4.10, 95% CI 1.84–9.15, *p* = 0.001).

## 4. Discussion

Carbapenem-resistant *Enterobacterales* (CRE) infections represent a critical challenge to global healthcare systems due to their association with high morbidity, mortality, and limited therapeutic options. This retrospective observational study highlights the significant clinical burden of CRE infections, with findings that align with and expand upon existing research in this area.

Recurrence of CRE infection occurred in approximately 16.8% of involved patients, with recurrence-related readmissions accounting for 12.9%. The recurrence rate in our study was approximately double the reported CRE recurrence rate by Rebold et al. of 8.6%. [26]. The overall 30-day readmission rate observed was notable, with recurrent infections driving a substantial portion of hospital readmissions. These findings indicate a high burden and suboptimal management of CRE infections. In addition, our study identified diabetes mellitus as a significant risk factor for infection recurrence, consistent with previous studies that have highlighted diabetes, prolonged hospitalization, and recent antibiotic exposure as predisposing factors to recurrent infections [8,27]. These findings underscore the importance of targeted interventions, such as improved glycemic control and enhanced antimicrobial stewardship, to mitigate the risk of recurrence and associated readmissions in high-risk patients.

Regarding mortality rates as primary outcomes, the mortality rates were 22.8% at 14 days, 30.7% at 30 days, and 42.6% at 90 days, respectively, underscoring the severe consequences of CRE infections, consistent with previous reports of mortality ranging from 30% to 70% in severe cases, particularly in patients with bloodstream infections or pneumonia [2,5,6]. The 14-day mortality rate, which indicates more CRE infection as a mortality attributable risk factor compared to 30-day and 90-day mortality rates, was markedly high in our study, and even higher rates (between 28.1% and 35.9%) were previously reported in patients with CRE bloodstream infections [28,29,30].

Multiple investigated variables were shown to be possible mortality predictors in the univariable logistic regression, including bacteremia, pneumonia, hypotension, cardiac arrest, altered mental status, severe sepsis/septic shock, ICU admission, inotropic support, CRRT, and mechanical ventilation. Some of these predictors were also previously reported, including ICU admission, mechanical ventilation, old age, central line insertion, and prior carbapenem use [31]. However, the multivariate Cox regression analysis (Table 5) that included age, gender, and the Charlson comorbidity index, along with variables that were significant in the univariable analysis (i.e., bacteremia, pneumonia, ICU admission, inotropic support, CRRT, mechanical ventilation, severe sepsis or septic shock, hypotension, and altered mental status) identified only three mortality predictors, namely ICU admission, pneumonia, and hypotension were independent predictors of mortality in this study, findings that are consistent with prior research [32,33,34]. These factors reflect the systemic complications and organ dysfunction commonly associated with severe CRE infections.

The association of bloodstream infections or pneumonia with higher mortality compared to other types of CRE infection has been observed in other studies, where localized infections, such as UTIs, tend to have better outcomes due to their less invasive nature [10]. This observation was further reinforced by Kaplan–Meier survival analyses in our study, which demonstrated significantly worse survival among patients with bacteremia or pneumonia compared to other infection types.

The PBS has been used as a practical bedside tool to assess the severity of acute infections. It includes five variables: temperature, blood pressure, need for mechanical ventilation, cardiac arrest, and mental status, offering a combined measure of clinical instability. Higher scores, especially those above four, are linked to increased mortality in bloodstream infections and other severe infections [6,11,35]. In our study, patients who did not survive 90 days had significantly higher PBS values than survivors (3.3 ± 3.6 vs. 0.7 ± 1.9, *p* < 0.001). This emphasizes the score’s prognostic value not only in CRE bacteremia but also in severe non-bacteremic CRE infections, where it appears to maintain predictive power. The INCREMENT-CPE score expands on this by integrating acute severity with comorbidities and treatment factors. It assigns weighted points for severe sepsis or shock, a PBS of six or higher, infection outside the urinary or biliary tract, inappropriate empirical therapy, and a Charlson comorbidity index of three or more. Patients are then classified as low risk (≤7 points) or high risk (≥8 points), with studies showing differences in 30-day mortality between these groups [36,37]. In our cohort, the INCREMENT-CPE score also effectively distinguished outcomes, with those who died within 90 days having notably higher scores compared to survivors (8.7 ± 4.5 vs. 4.9 ± 3.1, *p* < 0.001). Importantly, this trend was observed in both bacteremic and non-bacteremic cases, supporting its broader usefulness in CRE infections.

The role of antibiotic therapy was another critical aspect of this study. Combination therapy, particularly colistin-based regimens, was associated with improved survival. Previous studies have similarly reported the benefits of combination therapy in managing CRE infections, particularly in critically ill patients with limited treatment options [3,38]. However, the widespread resistance to carbapenems, aminoglycosides, and fluoroquinolones observed in this study reflects the global challenge of antimicrobial resistance, as highlighted by the World Health Organization’s designation of CRE as a critical priority pathogen [1]. This underscores the urgent need for new antibiotics and optimized therapeutic strategies to combat CRE infections.

This study has limitations that should be acknowledged. The retrospective design introduces the potential for selection bias, and missing or incomplete data may have affected the accuracy of some findings. Additionally, the study population was relatively small, and the research was conducted at a single medical center, which may limit the generalizability of the results to other settings. Despite these limitations, the findings contribute to the growing body of evidence on CRE infections and provide a foundation for future research.

## 5. Conclusions

This study highlights the substantial clinical burden of CRE in Eastern Saudi Arabia, with high recurrence, notable readmission rates, and mortality exceeding 40% at 90 days. Independent predictors of poor outcome included pneumonia, critical care admission, and hypotension, while diabetes mellitus emerged as a risk factor for recurrence. These findings underscore the importance of early recognition, risk stratification, and tailored management, supported by validated tools such as the Pitt bacteremia and INCREMENT-CPE scores. Strengthened infection control and stewardship measures, combined with vigilant monitoring of high-risk patients, remain essential. Future prospective, multi-center research should confirm these associations and evaluate novel therapeutic strategies in the vulnerable population.

## Figures and Tables

**Table 1:** Demographic and clinical characteristics of patients with CRE infections (*n* = 101).

Characteristics	Values
**Age** (Mean ± SD, years)	65.1 ± 17.1
**Gender**	
Male	43 (42.6%)
Female	58 (57.4%)
**Body weight** in kilograms (mean ± SD)	71.8 ± 18.2
**Isolated CRE pathogen**	
*Klebsiella pneumoniae*	95 (94.1%)
*Escherichia coli*	5 (5.0%)
*Serratia marcescens*	1 (1.0%)
**Type of CRE gene**	
OXA 48	71 (70.3%)
NDM	11 (10.9%)
OXA 48 and NDM	19 (18.8%)
**Type of CRE infection**	
Urinary tract infection	36 (35.6%)
Bacteremia	27 (26.7%)
Pneumonia	24 (23.8%)
Skin/soft tissue infection	9 (8.9%)
Wound infection	5 (5.0%)
Osteomyelitis	2 (2.0%)
Others	2 (2.0%)
**Source of infection**	
Hospital-acquired	79 (78.2%)
Community-acquired	22 (21.8%)
**Baseline renal function** (median and IQR)	
Serum creatinine (mg/dL)	1.2 (0.7–2.2)
Creatinine clearance (mL/min)	46 (29–97)
**Antibiotic combination therapy**	26 (26.5%)
CAZ-AVI-based combination	15 (15.3%)
Colistin-based combination	6 (6.1%)
Tigecycline-based combination	3 (3.1%)
Carbapenem-based combination	2 (2.0%)
**Antibiotic monotherapy**	72 (73.5%)
CAZ-AVI	41 (41.8%)
Colistin	6 (6.1%)
Tigecycline	5 (5.1%)
Carbapenems	15 (15.3%)
Fluoroquinolones	4 (4.1%)
Aminoglycosides	1 (1.0%)
**Antibiotic therapy duration** (median and IQR)	12 (7–18)

CAZ-AVI, Ceftazidime-avibactam; IQR, interquartile range; SD, standard deviation.

**Table 2:** Association of demographic variables, types of CRE pathogens, genes, and infections, sources of infection, and comorbidities with CRE infection recurrence and 90-day all-cause mortality.

	CRE Recurrence		*p*-Value	90-Day All-Cause Mortality		*p*-Value
	No (*n* = 84)	Yes (*n* = 17)		No (*n* = 58)	Yes (*n* = 43)	
**Age** (Mean ± SD, years)	65.0 ± 17.1	65.5 ± 17.6	0.902	63.2 ± 17.5	67.5 ± 16.5	0.216
**Gender**						
Male	37 (44.0%)	6 (35.3%)	0.506	23 (39.7%)	20 (46.5%)	0.491
Female	47 (56.0%)	11 (64.7%)		35 (60.3%)	23 (53.5%)	
**Isolated CRE pathogen**						
*Klebsiella pneumoniae*	78 (92.9%)	17 (100.0%)	0.655	54 (93.1%)	41 (95.3%)	0.261
*Escherichia coli*	5 (6.0%)	0 (0.0%)		4 (6.9%)	1 (2.3%)	
*Serratia marcescens*	1 (1.2%)	0 (0.0%)		0 (0.0%)	1 (2.3%)	
**Type of CRE gene**						
OXA 48	60 (71.4%)	11 (64.7%)	0.479	43 (74.1%)	28 (65.1%)	0.319
NDM	10 (11.9%)	1 (5.9%)		7 (12.1%)	4 (9.3%)	
OXA 48 and NDM	14 (16.7%)	5 (29.4%)		8 (13.8%)	11 (25.6%)	
**Type of CRE infection**						
Bacteremia	28 (33.3%)	5 (29.4%)	0.753	9 (15.5%)	24 (55.8%)	<0.001 *
Urinary tract infection	29 (34.5%)	7 (41.2%)	0.601	27 (46.6%)	9 (20.9%)	0.008 *
Pneumonia	21 (25.0%)	3 (17.6%)	0.756	8 (13.8%)	16 (37.2%)	0.006 *
Skin/soft tissue infection	7 (8.3%)	2 (11.8%)	0.645	8 (13.8%)	1 (2.3%)	0.074
Wound infection	5 (6.0%)	0 (0.0%)	0.586	4 (6.9%)	1 (2.3%)	0.391
Osteomyelitis	1 (1.2%)	1 (5.9%)	0.310	2 (3.4%)	0 (0.0%)	0.506
Others	2 (2.4%)	0 (0.0%)	>0.99	1 (1.7%)	1 (2.3%)	>0.99
**Source of infection**						
Hospital-acquired	68 (81.0%)	11 (64.7%)	0.195	43 (74.1%)	36 (83.7%)	0.249
Community-acquired	16 (19.0%)	6 (35.3%)		15 (25.9%)	7 (16.3%)	
**Comorbidities**						
Myocardial infarction	12 (14.3%)	1 (5.9%)	0.690	8 (13.8%)	5 (11.6%)	0.748
CHF	14 (16.7%)	5 (29.4%)	0.304	10 (17.2%)	9 (20.9%)	0.639
CVA or TIA	29 (34.5%)	5 (29.4%)	0.684	20 (34.5%)	14 (32.6%)	0.840
Dementia	10 (11.9%)	2 (11.8%)	>0.99	7 (12.1%)	5 (11.6%)	0.946
COPD	1 (1.2%)	0 (0.0%)	>0.99	1 (1.7%)	0 (0.0%)	>0.99
Peptic ulcer disease	5 (6.0%)	0 (0.0%)	0.586	2 (3.4%)	3 (7.0%)	0.648
Liver disease	1 (1.2%)	1 (5.9%)	0.310	0 (0.0%)	2 (4.7%)	0.179
Diabetes mellitus	50 (59.5%)	15 (88.2%)	0.024 *	36 (62.1%)	29 (67.4%)	0.577
Hemiplegia	1 (1.2%)	1 (5.9%)	0.310	0 (0.0%)	2 (4.7%)	0.179
Moderate to severe CKD	22 (26.2%)	5 (29.4%)	0.770	14 (24.1%)	13 (30.2%)	0.494
Solid tumor	5 (6.0%)	1 (5.9%)	>0.99	2 (3.4%)	4 (9.3%)	0.397
AIDS	1 (1.2%)	0 (0.0%)	>0.99	1 (1.7%)	0 (0.0%)	>0.99
**Charlson Comorbidity Index ^#^**	4.7 ± 2.6	5.3 ± 3.2	0.718	4.5 ± 2.8	5.2 ± 2.4	0.139

* *p*-value significant. AIDS, acquired immunodeficiency syndrome; CKD, chronic kidney disease; CHF, congestive heart failure; COPD, chronic obstructive pulmonary disease; CVA, cerebrovascular accident; SD, standard deviation; TIA, transient ischemic attack. # Charlson Comorbidity Index: a validated, weighted score that summarizes overall patient comorbidity burden.

**Table 3:** Associations of possible predisposing factors with CRE recurrence.

	**CRE Recurrence**		***p*-Value**
**Possible Predisposing Factors**	**No (*n* = 84)**	**Yes (*n* = 17)**	
	**N (%)**	**N (%)**	
**Previous hospitalization within 12 months**	62 (73.8%)	13 (76.5%)	>0.99
**Previous antibiotic exposures within 3 months**	72 (85.7%)	15 (88.2%)	>0.99
**Prolonged LOS (>14 days)**	63 (78.8%)	16 (94.1%)	0.183
**Antibiotic combination therapy**	22 (27.2%)	4 (23.5%)	>0.99
CAZ-AVI-based combination	12 (14.8%)	3 (17.6%)	0.721
Colistin-based combination	6 (7.4%)	0 (0.0%)	0.586
Tigecycline-based combination	2 (2.5%)	1 (5.9%)	0.439
Carbapenem-based combination	2 (2.5%)	0 (0.0%)	>0.99
**Antibiotic Monotherapy**	59 (72.8%)	13 (76.5%)	>0.99
CAZ-AVI	34 (42.0%)	7 (41.2%)	0.952
Colistin	4 (4.9%)	2 (11.8%)	0.278
Tigecycline	5 (6.2%)	0 (0.0%)	0.584
Carbapenems	12 (14.8%)	3 (17.6%)	0.721
Fluoroquinolones	3 (3.7%)	1 (5.9%)	0.539
Aminoglycosides	1 (1.2%)	0 (0.0%)	>0.99
**Duration of antibiotic therapy in days ^#^**	10 (7–18)	13 (10–19)	0.158

**^#^** Mean ± Standard deviation. CAZ-AVI, Ceftazidime-avibactam; LOS, length of stay.

**Table 4:** Associations of possible mortality predictors with 90-day all-cause mortality.

	90-Day All-Cause Mortality		
Possible Mortality Predictors	No (*n* = 58)	Yes (*n* = 43)	*p*-Value
	N (%)	N (%)	
Hypotension	8 (13.8%)	21 (48.8%)	<0.001 *
Cardiac arrest	3 (5.2%)	10 (23.3%)	0.007 *
Altered mental status	4 (6.9%)	17 (39.5%)	<0.001 *
Severe sepsis/septic shock	9 (15.5%)	21 (48.8%)	<0.001 *
ICU admission	18 (31.0%)	37 (86.0%)	<0.001 *
Inotropic support	2 (3.4%)	16 (37.2%)	<0.001 *
CRRT	2 (3.4%)	11 (25.6%)	0.001 *
Mechanical ventilation	8 (13.8%)	18 (41.9%)	0.001 *
Previous hospitalization within 12 months	43 (74.1%)	32 (74.4%)	0.975
Previous antibiotic exposures within 3 months	51 (87.9%)	36 (83.7%)	0.545
Prolonged LOS (>14 days)	47 (83.9%)	32 (78.0%)	0.462
**Pitt bacteremia score ^#^**	0.7 ± 1.9	3.3 ± 3.6	<0.001 *
**INCREMENT-CPE score ^#^**	4.9 ± 3.1	8.7 ± 4.5	<0.001 *
**Antibiotic combination therapy**	14 (24.6%)	12 (29.3%)	0.603
CAZ-AVI-based combination	6 (10.5%)	9 (22.0%)	0.121
Colistin-based combination	6 (10.5%)	0 (0.0%)	0.039 *
Tigecycline-based combination	1 (1.8%)	2 (4.9%)	0.570
Carbapenem-based combination	1 (1.8%)	1 (2.4%)	>0.99
**Antibiotic Monotherapy**	43 (75.4%)	29 (70.7%)	0.603
CAZ-AVI	24 (42.1%)	17 (41.5%)	0.949
Colistin	2 (3.5%)	4 (9.8%)	0.233
Tigecycline	4 (7.0%)	1 (2.4%)	0.396
Carbapenems	8 (14.0%)	7 (17.1%)	0.680
Fluoroquinolones	4 (7.0%)	0 (0.0%)	0.137
Aminoglycosides	1 (1.8%)	0 (0.0%)	>0.99
**Duration of antibiotic therapy in days ^#^**	12 (7–18)	10 (5–16)	0.257

**^#^** Mean ± Standard deviation. CAZ-AVI, Ceftazidime-avibactam; CRRT, Continuous Renal Replacement Therapy; ICU, Intensive Care Unit; LOS, length of stay. * *p* < 0.05 indicates statistical significance, meaning the observed differences are unlikely due to chance.

**Table 5:** Multivariate Cox regression analysis * of potential predictors of mortality in patients with CRE infection.

	Hazard Ratios	95% Confidence		*p*-Value
		Lower	Upper	
**Pneumonia**	2.39	1.23	4.64	0.010
**Critical care**	6.24	2.44	15.97	<0.001
**Hypotension**	4.10	1.84	9.15	0.001

* Multivariate Cox regression analysis was conducted using backward elimination. The included variables were age, gender, and the Charlson comorbidity index, along with those that were significant in the univariate analysis: bacteremia, pneumonia, critical care, inotropic support, continuous renal replacement therapy, mechanical ventilation, severe sepsis or septic shock, hypotension, and altered mental status.

## Data Availability

The raw data supporting the conclusions of this article will be made available by the authors on request.
